# Identification of the Natural Steroid Sapogenin Diosgenin as a Direct Dual-Specific RORα/γ Inverse Agonist

**DOI:** 10.3390/biomedicines10092076

**Published:** 2022-08-25

**Authors:** Patrik F. Schwarz, Alexander F. Perhal, Lucia N. Schöberl, Martin M. Kraus, Johannes Kirchmair, Verena M. Dirsch

**Affiliations:** Department of Pharmaceutical Sciences, Division of Pharmacognosy, University of Vienna, Josef-Holaubek-Platz 2, 1090 Vienna, Austria

**Keywords:** natural products, steroid sapogenin, diosgenin, anti-inflammatory, RORγ inverse agonists, RORα inverse agonists, retinoic acid-related orphan receptors, ROR, RORγ, RORα

## Abstract

The steroid sapogenin diosgenin is a well-known natural product with a plethora of described pharmacological activities including the amelioration of T helper 17 (Th17)-driven pathologies. However, the exact underlying mode of action of diosgenin leading to a dampened Th17 response is still largely unknown and specific molecular targets have yet to be identified. Here, we show that diosgenin acts as a direct ligand and inverse agonist of the nuclear receptor retinoic acid receptor (RAR)-related orphan receptor (ROR)α and RORγ, which are key transcription factors involved in Th17 cell differentiation and metabolism. IC_50_ values determined by luciferase reporter gene assays, employing constructs for either RORγ-Gal4 fusion proteins or full length receptors, were in the low micromolar range at around 2 µM. To highlight the functional consequences of this RORα/γ inverse agonism, we determined gene expression levels of important ROR target genes, i.e., *IL-17A* and glucose-6-phosphatase, in relevant cellular in vitro models of Jurkat T and HepG2 cells, respectively, by RT-qPCR (reverse transcription quantitative PCR). Thereby, it was shown that diosgenin leads to a dose-dependent decrease in target gene expressions consistent with its potent cellular ROR inverse agonistic activity. Additionally, in silico dockings of diosgenin to the ROR ligand-binding domain were performed to determine the underlying binding mode. Taken together, our results establish diosgenin as a novel, direct and dual-selective RORα/γ inverse agonist. This finding establishes a direct molecular target for diosgenin for the first time, which can further explain reported amendments in Th17-driven diseases by this compound.

## 1. Introduction

Retinoic acid receptor (RAR)-related orphan receptors (RORs) belong to the superfamily of ligand-dependent nuclear receptors with three subtypes expressed in humans: RORα, -β and -γ. While RORα and RORγ are widely expressed in peripheral tissues, e.g., liver, skeletal muscle or immune cells, the expression of RORβ is more restricted to the central nervous system. [1,2] Importantly, RORγ was shown to act as a key transcription factor in proinflammatory T helper 17 (Th17) cell differentiation, which can be induced downstream of the *signal transducer and activator of transcription* (STAT) 3 signaling pathway [3,4]. Furthermore, RORγ acts as a master regulator in hepatic gluconeogenesis, a central process in the development of metabolic disorders such as type 2 diabetes [5]. Consistent with these central roles in immunological and metabolic processes, RORγ represents an attractive molecular drug target, and it was shown that the inhibition of RORγ transcriptional activity is effective against Th17-mediated conditions [6,7,8] as well as metabolic pathologies. [5,9] Apart from RORγ, RORα was also shown to be indispensable for Th17 development as only deletions of both nuclear receptors completely abolished Th17 differentiation [10]. Furthermore, mice that express a truncated and, thus, dysfunctional mutant of RORα (*Rora^sg/sg^* “staggerer” mice) were shown to be resistant to diet-induced obesity [11], show impaired gluconeogenesis in the liver [12], and exhibit increased insulin sensitivity and glucose-uptake in skeletal muscles [13]. Notably, many target genes, such as glucose-6-phosphatase (*G6PC*), were shown to be mutually regulated by RORγ and RORα [14]. Therefore, RORα represents a promising drug target itself and can be considered as functionally redundant to RORγ as far as immunological [15] and metabolic [16,17,18] effects are concerned.

**Figure 1:**
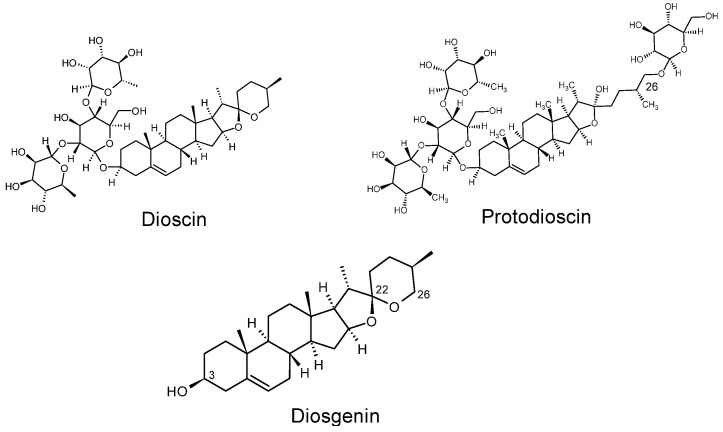
Structural formulas of the saponins dioscin and protodioscin (**top**) and of the sapogenin diosgenin (**bottom**).

Dioscoreae rhizoma (chinese yam, shānyào, lit. “mountain medicine”) is described in traditional Chinese medicine to tonify the Qi of lung, spleen and kidney and to nourish the kidney’s essence [19]. Diosgenin was first discovered in 1937 by Tsukamoto and colleagues in *Dioscorea tokoro* (Dioscoreaceae) and can be found, along with its corresponding saponins dioscin and protodioscin, in many different *Dioscorea* species [20,21]. Chemically, diosgenin belongs to the group of steroid sapogenins consisting of a spirostan scaffold with a characteristic spiroketal in position 22 and a hydroxy group in position 3 (Figure 1, bottom), which is glycosylated in the corresponding saponins dioscin and protodioscin with a trisaccharide (Figure 1, top). The saponin protodioscin is additionally glycosylated in position 26, resulting in the formation of a hemiketal (Figure 1, top right).

Starting in the 1940s, diosgenin gained considerable attention due to its versatile use as a precursor compound for the synthesis of many important steroid hormones, including progesterone described by Russell Marker [22] or cortisone by Carl Djerassi [23]. Meanwhile, it became evident that diosgenin exerts many different beneficial pharmacological activities itself, including antiproliferative, anti-inflammatory, lipid-lowering and hypoglycemic activities (reviewed in [24,25]). Notably, several recent studies reported beneficial effects of diosgenin and its saponin dioscin in Th17-mediated diseases such as experimental autoimmune encephalomyelitis [26] or collagen-induced arthritis [27,28,29] where they could effectively suppress Th17 cell differentiation and ameliorate disease severities. Thus far, these studies attributed these beneficial effects to a reduction in STAT3 expression levels [26], its phosphorylation state [28] and/or to reduced expression levels of the downstream transcription factor RORγ [27,29]. Furthermore, in some studies diosgenin effectively reduced the protein expression of the RORγ target gene interleukin (IL)-17 [3] both in vitro and in vivo. [29,30] These particular studies were performed in isolated primary murine T lymphocytes from collagen-induced arthritis (CIA) or experimental autoimmune encephalomyelitis (EAE) disease models as well as in a keratinocyte (HaCaT) cell line. [29,30] Taken together, the data collected in previous studies led us to the hypothesis that diosgenin may not only act via reduction of RORγ expression as already demonstrated before (e.g., by inhibition of STAT3 signaling) but also as a direct ligand of RORγ. This premise was further fueled by the high structural similarity between diosgenin and other reported natural RORγ inhibitors (reviewed in [31]).

**Figure 2:**
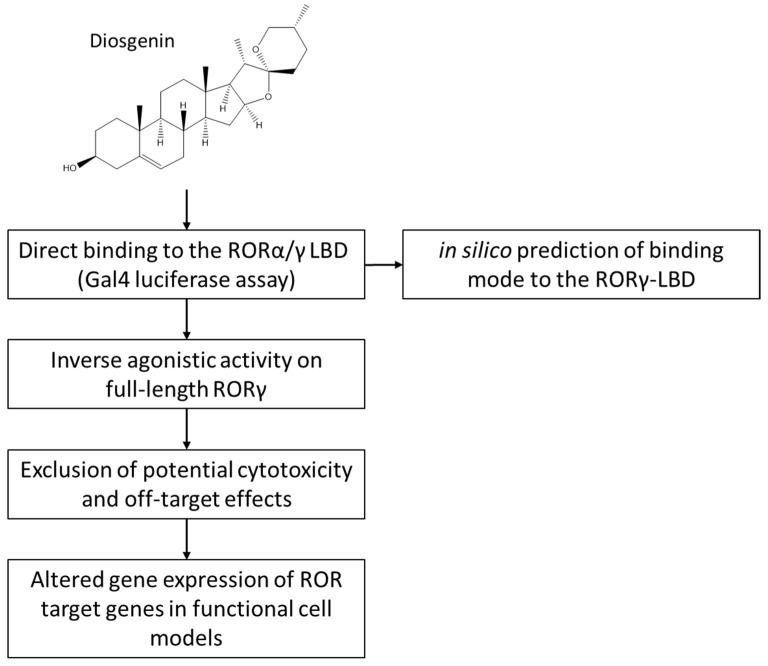
Graphical scheme of the study approach. First, the direct binding of diosgenin to the RORγ (and subsequently the RORα) LBD was examined using RORα/γ-Gal4 luciferase assays. The inverse agonistic activity elucidated via the Gal4 assays was subsequently confirmed using full-length RORγ luciferase assays. Then, cytotoxicity and potential off-target effects were excluded using resazurin and nuclear receptor-Gal4 assays, respectively. Finally, changes in target gene expression of RORγ target genes in functional cellular models were studied. In silico prediction of diosgenin’s binding mode was performed by employing molecular docking.

To answer this question and to gain a better overall understanding of the pharmacology of diosgenin, we studied its effects on RORγ using in vitro and in silico techniques (Figure 2).

## 2. Materials and Methods

### 2.1. Cell Lines, Plasmids and Chemicals

Human embryonic kidney 293 (HEK293) and hepatoma G2 (HepG2) cells were purchased from the American Type Culture Collection (ATCC, Manassas, VA, USA). Jurkat T cells were a kind gift from Manfred Ogris (Department of Pharmaceutical Sciences, University of Vienna, Vienna, Austria). Dulbecco’s modified Eagle medium (DMEM), Eagle’s minimum essential medium (EMEM), Roswell Park Memorial Institute Medium-1640 (RPMI-1640), L-glutamine and penicillin–streptomycin mixtures were obtained from Lonza (Basel, Switzerland). Fetal bovine serum (FBS) was acquired from biowest (Nuaillé, France). Trypsin, the High-Capacity cDNA Reverse Transcription Kit, Lipofectamine LTX Reagent with PLUS Reagent, and Lipofectamin 3000 were purchased from Thermo Fisher Scientific (Waltham, MA, USA). Enhanced green fluorescent protein (pEGFP-N1) was obtained from Clontech (Mountain View, CA, USA). All other plasmids—the RORγ-ligand-binding-domain (LBD)-Gal4-DNA-binding domain fusion construct (RORγ-Gal4), tk(MH1000)4xLuc, farnesoid X receptor (FXR)-Gal4, liver X receptor (LXR)-α/β-Gal4, peroxisome proliferator-activated receptor (PPAR)-γ-Gal4, retinoid X receptor (RXR)-α/β-Gal4, murine retinoic acid receptor (mRAR)-α-Gal4, RORα/β-Gal4, full-length RORγ transcript variant 1 (RORγV1) and ROR-response element (RORE)-Luc—were kindly made available by providers listed in Table S1. Primers for IL-17A and G6PC were synthesized at Microsynth (Balgach, Switzerland). Primers for Glyceraldehyde 3-phosphate dehydrogenase (GAPDH) and beta actin were acquired from Qiagen (Hilden, Germany). The Cell Activation Cocktail (without Brefeldin A) was purchased from Bio-Legend (San Diego, CA, USA). The innuPREP RNA Mini Kit 2.0 was obtained from Analytik Jena (Jena, Germany). The GoTaq Green Master Mix and the 5X reporter lysis buffer were purchased from Promega (Fitchburg, WI, USA). Ethanol (EtOH) 96% and ethylenediaminetetraacetic acid (EDTA) were obtained from Carl Roth (Karlsruhe, Germany). Diosgenin, SR2211, resazurin sodium salt, digitonin, T0901317, GW4064, GW3965, rosiglitazone, bexarotene and all-trans retinoic acid (ATRA) were acquired from Sigma-Aldrich (St. Louis, MO, USA). Dioscin and protodioscin were purchased from Biomol (Hamburg, Germany). Catalog numbers of all commercially obtained materials are listed in Table S2.

### 2.2. Cell Culture

HEK293 cells, Jurkat T cells and HepG2 cells were cultured in complete DMEM, RPMI-1640 and EMEM, respectively, which were supplemented with 10% FBS, 2 mM glutamine, 100 U/mL penicillin and 100 µg/mL streptomycin at 37 °C and 5% CO_2_. Cells were passaged every 2–3 days and only used up to passage number 30. Cell number and viability were determined using an automated cell counter (Vi-CELL™ XR Cell Viability Analyzer, Beckmann Coulter GmbH, Krefeld, Germany). For some experiments, media supplemented with 5% charcoal-stripped FBS (stripped media) were used.

### 2.3. Luciferase Assays

For luciferase assays, 6 × 10^6^ HEK293 cells were seeded on 150 mm cell culture dishes and incubated for five hours. Afterwards, cells were transfected by calcium phosphate co-precipitation using 5 µg of the nuclear receptor (RORγ-Gal4 or full-length RORγ), 5 µg of a luciferase reporter (tk(MH1000)4xLuc for RORγ-Gal4 experiments or RORE-Luc for full-length RORγ experiments) and 3 µg pEGFP-N1 (for assessing transfection efficiency) plasmid DNA and incubated overnight.

For selectivity tests, 5 µg of different nuclear receptor-Gal4 constructs (RORα/β, FXR, LXRα/β, PPARγ, RXRα/β and mRARα) were transfected together with tk(MH1000)4 × Luc and eGFP, as described before.

On the following day, the medium was changed, and cells were further incubated for another four to five hours before adding trypsin/EDTA to detach them from dishes. Complete DMEM was added to stop trypsinization, and cell suspensions were centrifuged at 410× *g* for four minutes. Cell pellets were resuspended in stripped DMEM and seeded at a density of 5 × 10^4^ viable cells per well in a 96-well plate before treatment with the vehicle control (0.096% EtOH), respective positive controls or diosgenin at the indicated concentrations for 18 h. Subsequently, cells were lysed using 5X reporter lysis buffer and luminescence and fluorescence values were measured using a spectrophotometer (Tecan Group AG, Männedorf, Switzerland). Relative luminescence units (RLU) were normalized to relative fluorescence units (RFU) and subsequently normalized to the vehicle control EtOH 0.096% (set to 1.0). Results are expressed as fold activations relative to the vehicle control.

### 2.4. Resazurin Conversion Assay

To check the metabolic activity of cells correlating with cell viability, cells were treated with the vehicle control (EtOH), digitonin at 50 µg/mL (positive control for cytotoxicity) or test compounds (diosgenin, dioscin or protodioscin) at the indicated concentrations for 18 h. The next day, the medium was carefully removed and replaced with stripped DMEM containing 10 µg/mL resazurin sodium salt. Cells were incubated for five hours before RFU values were measured at λ_em_ = 590 nm using a spectrophotometer.

### 2.5. Determination of Target Gene Expression by RT-qPCR

For reverse transcription quantitative PCR (RT-qPCR) experiments, 3 × 10^5^ Jurkat T cells or 5 × 10^5^ HepG2 cells per well were seeded on a 48 or a 6-well plate, respectively. For Jurkat T cells, 0.5 µg of full-length RORγ plasmids were transfected using Lipofectamine LTX Reagent with PLUS Reagent according to the manufacturer’s instructions. For HepG2 cells, 2.5 µg of full-length RORγ plasmids were transfected using Lipofectamine 3000 according to the manufacturer’s instructions. After 24 h incubation, transfected cells were treated with vehicle control (0.096% EtOH), positive control (1 µM SR2211 [7]) or diosgenin at the indicated concentrations. HepG2 cells were treated with compounds for 6 h. Jurkat T cells were stimulated for cytokine production after 18 h compound treatment using a cell activation cocktail containing phorbol-12-myristate-13-acetate and ionomycin at optimized concentrations for 5 h. Total RNA was extracted from cells using the innuPREP RNA Mini Kit 2.0 according to manufacturer’s instructions. The concentration and purity of isolated RNA were measured using a spectrophotometer (Thermo Fisher Scientific, Waltham, MA, USA), and aliquoted RNAs were stored at −70 °C until use. 1 µg RNA was reverse transcribed into cDNA using the High-Capacity cDNA Reverse Transcription Kit according to manufacturer’s instructions. Primer sequences for RT-qPCR amplifications are provided in Table 1.

RT-qPCR amplification reactions were performed using the GoTaq Green Master Mix with a cDNA amount of 40 ng and 10 µM primer concentration (or diluted as recommended by the manufacturer in case of QuantiTect primer) in a final reaction volume of 15 µL on a thermocycler (Roche Diagnostics, Rotkreuz, Switzerland). The PCR reaction consisted of one initial denaturation step (2 min at 95 °C) and 50 amplification cycles (denaturation step: 15 s at 95 °C, annealing/extension step: 1 min at 60 °C). Expression levels of target genes were calculated using the 2^−ΔΔCt^ method [33] whereby the target gene expression levels were first normalized to the expression levels of the control housekeeping genes *GAPDH* or *beta actin* before normalization to the expression levels of the vehicle control.

### 2.6. In Silico Docking

An X-ray structure of the RORγ-LBD in complex with ursonic acid (PDB: 6J3N; resolution 1.99 Å) was selected for docking with GOLD (software version v2020.2.0, Cambridge Crystallographic Data Centre, Cambridge, UK) [34,35]. The protein structure was prepared with the Protein Preparation Wizard within the Maestro molecular modeling environment (software version 2021-3, Schrödinger Inc., New York, NY, USA) [36]. This preparation procedure included (i) the addition of hydrogen atoms as well as the assignment of protonation and metal charge states with Epik (a software module integrated into Maestro), (ii) the sampling of water orientations and the optimization of the hydrogen bond network and (iii) a restrained minimization using the OPLS3e force field [37] in order to converge heavy atoms to an RMSD of 0.30 Å (default settings used for all operations). A 3D structure of diosgenin was prepared with LigPrep within Maestro using default settings.

For docking with GOLD, the ligand-binding site was defined within GOLD to contain any atoms located within 6 Å of the co-crystallized ligand ursonic acid. Water molecules 701 and 756 were allowed to toggle on and off and to spin during docking. Default settings were used for the genetic algorithm, and a total of ten docking runs were executed (resulting in ten ligand poses). ChemPLP was used as scoring function.

### 2.7. Statistical Analysis

All data are presented as mean values ± standard deviation (SD). Data were analyzed for normal distribution by a Shapiro–Wilk test. For normally distributed data, one-way analysis of variance (ANOVA) followed by Dunnett’s post hoc test was performed to determine statistical significance between treatment groups and the vehicle control group. For non-normally distributed data, a non-parametric Kruskal–Wallis test followed by Dunn’s post hoc test was performed. *p*-values ≤ 0.05 were considered to be statistically significant.

Concentration–response curves were fitted by nonlinear regression with sigmoidal dose–response curves and a standard Hill coefficient of −1.0. Effects were considered as concentration-dependent when fitting of a sigmoidal regression curve was reasonably possible as determined by a goodness-of-fit value (R^2^) close to 1.0. All statistical evaluations were performed with GraphPad Prism (software version 9.4, GraphPad Software Inc., San Diego, CA, USA).

## 3. Results

### 3.1. Diosgenin Is a Potent and Direct Inverse Agonist of the Nuclear Receptor RORγ

**Figure 3:**
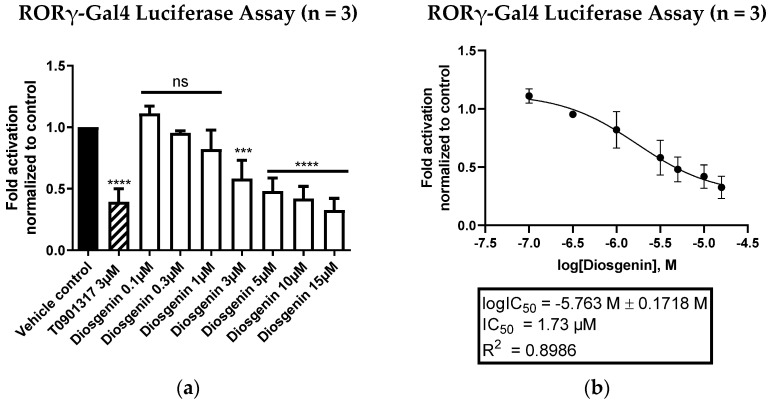
RORγ-Gal4 luciferase assay of diosgenin in HEK293 cells. (**a**) Diosgenin was tested at different concentrations in a cell-based RORγ-Gal4 luciferase assay to determine its inverse agonistic activity on RORγ. The luminescence signals derived from the luciferase reporter were normalized to eGFP fluorescence and expressed as fold activation normalized to the vehicle control (0.096% EtOH). The described RORα/γ inverse agonist T0901317 (3 µM) was used as a positive control. Bar charts represent transactivation activities expressed as mean ± SD of three biological replicates (n = 3) measured in technical quadruplicates. One-way ANOVA followed by Dunnett’s post hoc test were used for statistical analysis. **** *p* ≤ 0.0001, *** *p* ≤ 0.001, ns *p* > 0.05 compared to vehicle control. (**b**) Fitted concentration–response curve of the inverse agonistic activity of diosgenin on RORγ-Gal4 with a determined IC_50_ value of 1.73 µM. The curve was fitted by nonlinear regression using a standard Hill coefficient of −1.0. Data are presented as means ± SD of three biological replicates (n = 3) measured in technical quadruplicates.

In order to evaluate the potential of diosgenin as a direct RORγ inverse agonist, luciferase assays employing RORγ-Gal4 were performed. Thereby, diosgenin showed a potent and concentration-dependent inverse agonistic activity with a determined IC_50_ value of 1.73 µM, which is highly comparable to that of the described RORα/γ inverse agonist T0901317 [38] (Figure 3).

**Figure 4:**
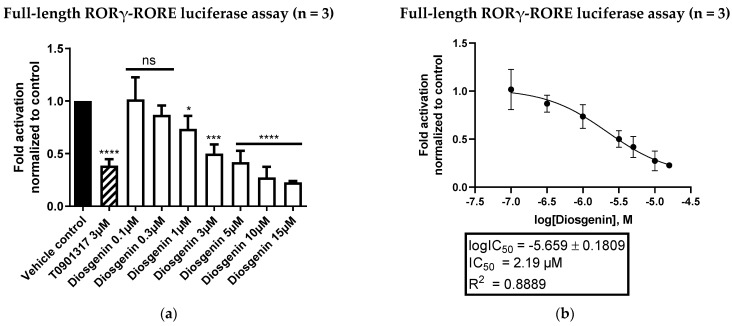
Full-length RORγ-RORE luciferase assay in HEK293 cells. (**a**) Diosgenin was tested at different concentrations in a cell-based full-length RORγ luciferase assay to determine its inverse agonistic activity on full-length RORγ. The luminescence signals derived from the luciferase reporter were normalized to eGFP fluorescence and expressed as fold activation normalized to the vehicle control (0.096% EtOH). The described RORα/γ inverse agonist T0901317 (3 µM) was used as a positive control. Bar charts represent transactivation activities expressed as mean ± SD of three biological replicates (n = 3) measured in technical quadruplicates. One-way ANOVA followed by Dunnett’s post hoc test were used for statistical analysis. **** *p* ≤ 0.0001, *** *p* ≤ 0.001, * *p* ≤ 0.05, ns *p* > 0.05 compared to vehicle control. (**b**) Fitted concentration–response curve of the inverse agonistic activity of diosgenin on full-length RORγ with a determined IC_50_ value of 2.19 µM. The curve was fitted by nonlinear regression using a standard Hill coefficient of −1.0. Data are presented as means ± SD of three biological replicates (n = 3) measured in technical quadruplicates.

To further confirm the diosgenin-mediated reduction in RORE-dependent transcriptional activity, a luciferase assay employing full-length RORγ (RORγV1) and a luciferase reporter gene under control of RORE was performed. Figure 4 shows the inverse agonistic activity of diosgenin on RORE-dependent transcriptional activity in a dose-dependent manner with a determined IC_50_ value of 2.19 µM.

### 3.2. Diosgenin Shows No Signs of Cytotoxicity in a Resazurin Conversion Assay

**Figure 5:**
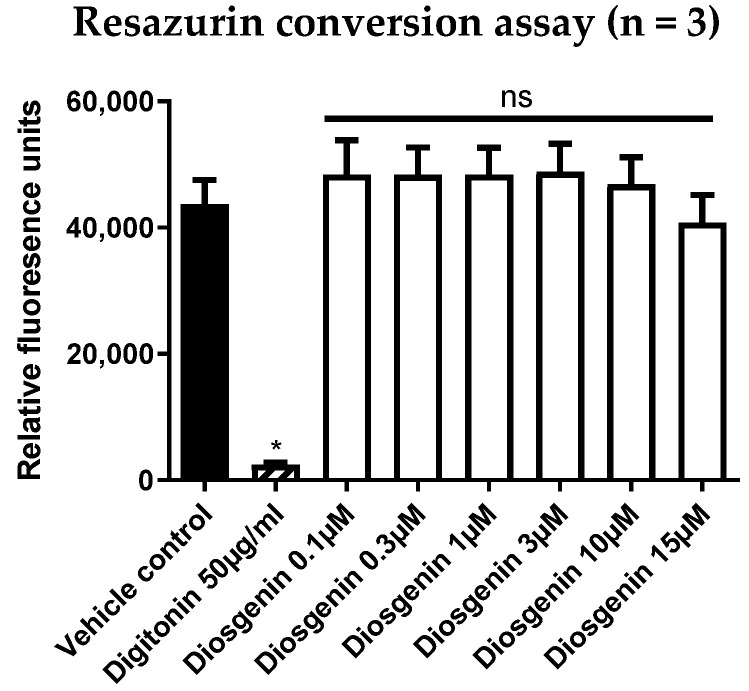
Resazurin conversion assay of diosgenin in HEK293 cells. To exclude potential cytotoxic effects of diosgenin in HEK293 cells, a resazurin conversion assay was performed. Cells were treated with digitonin (50 µg/mL) as a positive control or diosgenin at the indicated concentrations for 18 h. After the addition of resazurin (10 µg/mL), cells were incubated for another 5 h before RFU values were measured at λ_em_ = 590 nm. Data are presented as means ± SD of three biological replicates (n = 3) measured in technical quadruplicates. Kruskal–Wallis tests followed by Dunn’s post hoc test were used for statistical analysis. * *p* ≤ 0.05, ns *p* > 0.05 compared to the vehicle control.

To exclude the possibility that the observed inverse agonistic activity of diosgenin might be biased by the cytotoxicity of the compound, we performed resazurin conversion assays in HEK293 cells treated with increasing concentrations of diosgenin. Thereby, no cytotoxicity of diosgenin could be observed up to the highest concentration (15 µM) used for all assays (Figure 5). Additionally, no morphological signs of cytotoxicity could be observed microscopically in diosgenin-treated cells after overnight incubation (data not shown).

### 3.3. The Steroid Saponins Dioscin and Protodioscin Do Not Act as ROR Inverse Agonists in Non-Toxic Concentrations

**Figure 6:**
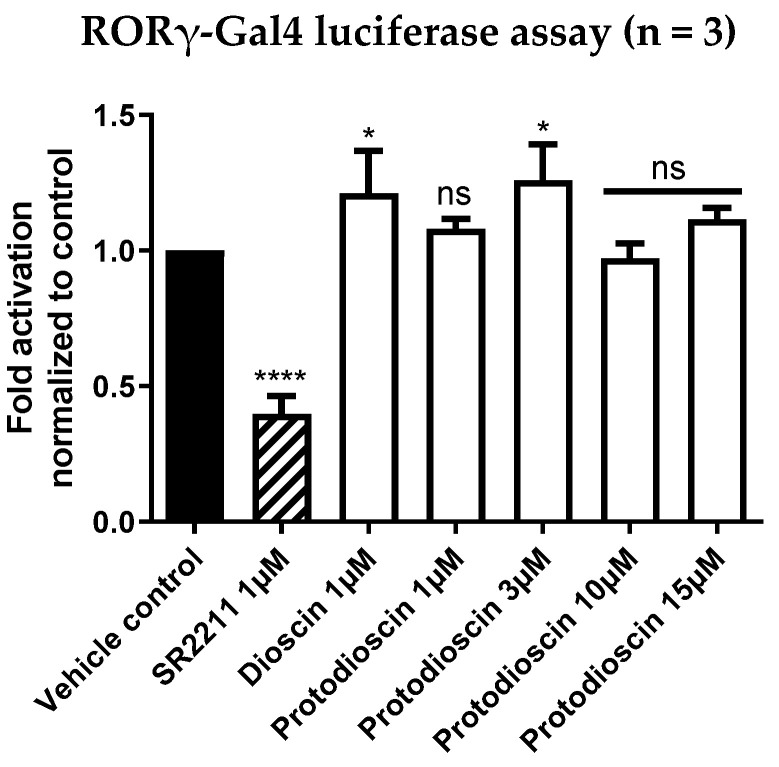
RORγ-Gal4 luciferase assay of dioscin and protodioscin in HEK293 cells. Dioscin and protodioscin were tested at non-cytotoxic concentrations in a cell-based RORγ-Gal4 luciferase assay to determine their potential activities on RORγ. The luminescence signals derived from the luciferase reporter were normalized to eGFP fluorescence and expressed as fold activation normalized to the vehicle control (0.096% EtOH). The described RORγ inverse agonist SR2211 (1 µM) was used as a positive control. Bar charts represent transactivation activities expressed as mean ± SD of three biological replicates (n = 3) measured in technical quadruplicates. One-way ANOVA followed by Dunnett’s post hoc test were used for statistical analysis. **** *p* ≤ 0.0001, * *p* ≤ 0.05, ns *p* > 0.05 compared to vehicle control.

In a next step, we investigated whether the corresponding saponins of diosgenin, dioscin and protodioscin (Figure 1, top) were also able to act on RORγ as inverse agonists. Therefore, both saponins were tested in RORγ-Gal4 luciferase assays, as described above. Thereby, dioscin exhibited strong cytotoxicity in HEK293 cells up to a concentration of 1 µM (Figure S1), a concentration at which no inverse agonistic activity on RORγ could be observed (Figure 6). On the other hand, protodioscin showed no apparent cytotoxicity in HEK293 cells (Figure S1) but also did not show any inverse agonistic activity on RORγ at any of the concentrations tested (Figure 6).

### 3.4. Diosgenin Is a Dual Specific Inhibitor of RORα and RORγ, but Shows No Activity on RORβ or Other Important Nuclear Receptors

**Figure 7:**
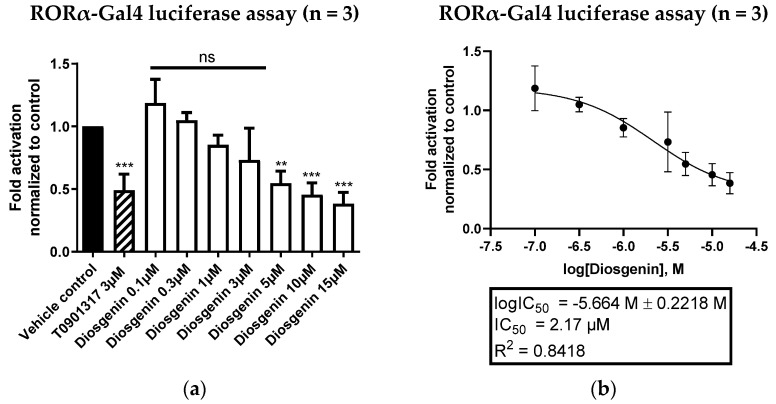
RORα-Gal4 luciferase assay of diosgenin in HEK293 cells. (**a**) Diosgenin was tested at different concentrations in a cell-based RORα-Gal4 luciferase assay to determine its inverse agonistic activity on RORα. The luminescence signals derived from the luciferase reporter were normalized to eGFP fluorescence and expressed as fold activation normalized to the vehicle control (0.096% EtOH). The described RORα/γ inverse agonist T0901317 (3 µM) was used as a positive control. Bar charts represent transactivation activities expressed as mean ± SD of three biological replicates (n = 3) measured in technical quadruplicates. One-way ANOVA followed by Dunnett’s post hoc test were used for statistical analysis. *** *p* ≤ 0.001, ** *p* ≤ 0.01, ns *p* > 0.05 compared to vehicle control. (**b**) Fitted concentration–response curve of the inverse agonistic activity of diosgenin on RORα-Gal4 with a determined IC_50_ value of 2.17 µM. The curve was fitted by nonlinear regression using a standard Hill coefficient of −1.0. Data are presented as means ± SD of three biological replicates (n = 3) measured in technical quadruplicates.

To examine whether diosgenin is a selective modulator of ROR receptors, we performed additional luciferase screenings by employing several other important nuclear receptor-Gal4 constructs, including FXR, LXRα, LXRβ, PPARγ, RXRα, RXRβ and mRARα. Thereby, diosgenin showed no activities on any other nuclear receptor-Gal4 construct at the tested concentrations (Figure S2). To further investigate the ROR subtype selectivity of diosgenin, luciferase assays with RORα and RORβ were performed as well. Notably, the results obtained showed a lack of activity on RORβ (Figure S2), while diosgenin was identified as an equipotent inverse agonist of RORα with an IC_50_ value of 2.17 µM (Figure 7). This indicates that diosgenin acts as a dual specific inverse agonist of RORα and RORγ while showing high selectivity over RORβ and other nuclear receptors that bind ligands of the sterol metabolism, such as LXRs or FXR [39].

### 3.5. Diosgenin Downregulates ROR Target Gene Expression in Functional Cellular Models

**Figure 8:**
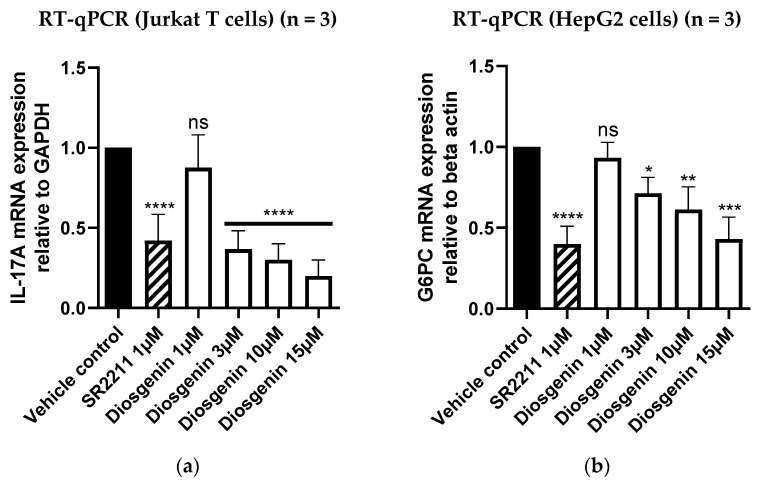
Determination of target gene expression in diosgenin-treated Jurkat T and HepG2 cells by RT-qPCR. To investigate the consequences of ROR inverse agonism by diosgenin, mRNA expression levels of RORγ-regulated target genes were determined by RT-qPCR in functional cellular models (**a**) Downregulation of the RORγ target gene *IL-17A* in RORγ-transfected Jurkat T cells after treatment with diosgenin at different concentrations. The expression levels of *IL-17A* were normalized to the expression levels of the housekeeping gene *GAPDH* and subsequently normalized to the vehicle control (0.096% EtOH). The described RORγ inverse agonist SR2211 (1 µM) was used as a positive control. Bar charts represent expression levels relative to vehicle control expressed as mean ± SD of three biological replicates (n = 3) measured in technical triplicates. One-way ANOVA followed by Dunnett’s post hoc test were used for statistical analysis. **** *p* ≤ 0.0001, ns *p* > 0.05 compared to vehicle control (**b**) Downregulation of the RORγ target gene *G6PC* in RORγ-transfected HepG2 cells after treatment with diosgenin at different concentrations. The expression levels of *G6PC* were normalized to the expression levels of the housekeeping gene *beta actin* and subsequently normalized to the vehicle control (0.096% EtOH). The described RORγ inverse agonist SR2211 (1 µM) was used as a positive control. Bar charts represent expression levels relative to vehicle control expressed as mean ± SD of three biological replicates (n = 3) measured in technical triplicates. One-way ANOVA followed by Dunnett’s post hoc test were used for statistical analysis. **** *p* ≤ 0.0001, *** *p* ≤ 0.001, ** *p* ≤ 0.01, * *p* ≤ 0.05, ns *p* > 0.05 compared to vehicle control.

In order to verify the functional consequences of RORα/γ inhibition by diosgenin on the gene expression of important ROR-regulated target genes in suitable cell models, RT-qPCR experiments were performed. As RORγ is a key transcription factor involved in Th17 cell differentiation and the production of their signature cytokine IL-17A [40], the expression levels of *IL-17A* were determined in RORγ-transfected Jurkat T cells upon treatment with diosgenin at different concentrations. Jurkat T cells represent a human leukemia T cell line, which was shown to express RORγ and IL-17A previously [41] and was, therefore, chosen as a suitable cell model for investigations of altered ROR-mediated IL-17A production in this study. Concomitant with its RORα/γ inverse agonism, the treatment with diosgenin led to a significant reduction in *IL-17A* mRNA expression in this cell model (Figure 8a).

To further confirm the functional activity of diosgenin on another important ROR target gene, mRNA transcripts of glucose-6-phosphatase (*G6PC*) [5] were quantified in RORγ-transfected HepG2 cells by RT-qPCR. As gluconeogenesis is a metabolic process mainly occurring in the liver, the hepatocellular HepG2 cell line was chosen as a suitable cell model for these experiments. Consistently, treatment of these cells with diosgenin led to a significant reduction in *G6PC* expression levels (Figure 8b).

To further exclude potential cytotoxicity of diosgenin in these two cell lines, resazurin conversion assays were performed and no significant reductions in cell viability could be detected up to a concentration of 15 µM (Figure S3).

### 3.6. In Silico Modelling of the Binding Mode of Diosgenin to the Ligand-Binding Domain of RORγ

**Figure 9:**
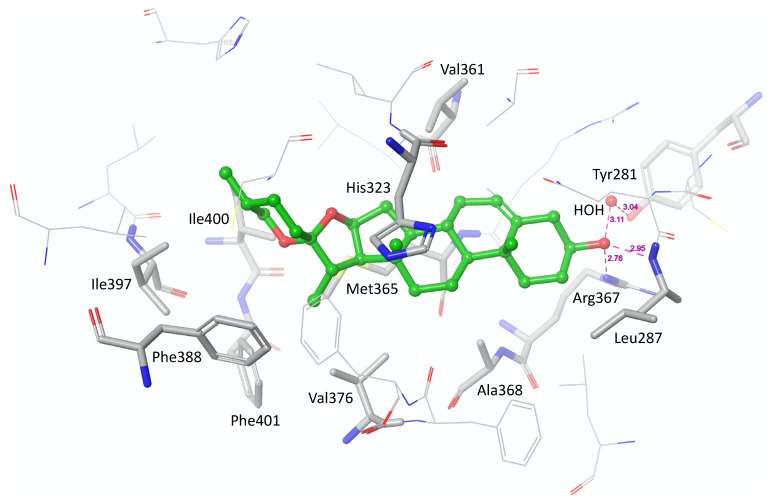
Predicted binding mode of diosgenin (green carbon atoms) in the RORγ ligand-binding domain. To elucidate the binding mode of diosgenin to the RORγ-LBD, molecular docking was employed. Protein preparation was performed using Maestro, and subsequent docking was performed with GOLD. Diosgenin is predicted to form hydrophobic interactions with residues Leu287, His323, Val361, Met365, Ala368, Val376, Phe388, Ile397, Ile400 and Phe401 of the RORγ ligand-binding domain. The hydroxy group in position 3 forms hydrogen bonds with Leu287, Arg367 and, via a water molecule, with Tyr281. Amino acid residues forming hydrophobic interactions or hydrogen bonds with the ligand diosgenin are marked by the thick tube representation. The predicted hydrogen bonds are indicated with purple, dashed lines.

The likely binding mode of diosgenin in the RORγ-LBD was derived by in silico docking with GOLD [34]. A total of ten docking poses were generated. All generated poses are consistent with respect to the location and orientation of the ligand, and the docking scores indicate a good geometric match of the predicted poses and the ligand-binding site (ChemPLP scores between 76.671 and 76.858). The docking poses indicate that the binding of diosgenin is likely driven by hydrophobic interactions with multiple residues forming the ligand-binding site, including Leu287, His323, Val361, Met365, Ala368, Val376, Phe388, Ile397, Ile400 and Phe401. The hydroxy moiety in position 3 of diosgenin is predicted to act as the key anchoring group by forming hydrogen bond interactions with Leu287, Arg367 and, via a water molecule, with Tyr281 (Figure 9).

## 4. Discussion

Diosgenin represents a natural steroid sapogenin with a plethora of pharmacological activities, including anti-inflammatory and anti-diabetic effects demonstrated previously both in vitro and in vivo (recently reviewed in [40]). However, the specific underlying molecular targets regulated by diosgenin that are responsible for these effects remain largely unknown and have yet to be identified. In this regard, we could show, for the first time, that diosgenin acts as a direct and potent inverse agonist of the nuclear receptors RORα and RORγ. The activity of diosgenin could be demonstrated in this study by showing a direct binding and transcriptional repression of RORα/γ in both luciferase assays as well as by evaluating altered target gene expressions in functional cell models. Additionally, an in silico predicted binding mode of diosgenin to the RORγ-LBD was proposed, and potentially limiting cytotoxic effects were excluded. These findings add considerable knowledge to the underlying mode of action of diosgenin, especially in the context of its previously described beneficial effects in T cell-related inflammatory diseases. Specifically, its observed beneficial effects in Th17 cell-mediated ailments such as EAE or rheumatic arthritis, can be further explained by the ROR inverse agonistic activity shown in this study. Together with previously identified effects on the STAT3 signaling pathway and observed reductions in the expression levels of RORγ [27,29,42], the results of this study contribute to a more comprehensive understanding of diosgenin’s molecular actions in these conditions. Moreover, diosgenin could be identified in this study as a dual specific inverse agonist of both RORα and RORγ while showing high specificity over other important nuclear receptors, including RORβ. This is of particular interest as RORα was shown to be essential for Th17 cell differentiation on its own and was recently confirmed to be a non-redundant factor for pathogenic Th17 function [10]. Therefore, RORα was proposed as an alternative target for the treatment of Th17-associated diseases [43].

Interestingly, we found that the steroid saponins dioscin and protodioscin showed no activity on RORγ at non-cytotoxic concentrations in luciferase assays, limiting this activity to the aglycon diosgenin. However, it has been shown that a high proportion of ingested dioscin can become hydrolyzed to diosgenin at low pH values of gastric acid in the stomach [44]. This can also explain why dioscin was shown to be capable of inhibiting Th17 cell differentiation in vivo when applied orally [27,28] despite the lack of in vitro activity on RORγ in our study.

In additional experiments, we could confirm that diosgenin was also able to downregulate the expression of the RORα/γ target genes *IL-17A* and glucose-6-phosphatase (*G6PC)* in suitable cellular models, further highlighting the functional consequences of the ROR inverse agonism. However, a potential limitation of these findings is the lack of confirmation on protein levels in this study, although other studies could already confirm such effects of diosgenin on protein levels for IL-17 in other cell models [29,30]. Moreover, alterations in mRNA expression levels represent the closest functional consequence of compounds acting as nuclear receptor modulators detectable within cells. Nevertheless, further confirmations of these effects on protein levels are needed and should be addressed in follow-up studies.

Eventually, molecular docking revealed that diosgenin binding to the RORγ-LBD is likely driven by hydrophobic interactions as well as several anchoring hydrogen bonds formed by the hydroxyl group in position 3 of diosgenin. This might explain the observed inactivity of the steroid saponins dioscin and protodioscin on RORγ as both of them are glycosylated at position 3. To experimentally verify the importance of the hydroxyl moiety in position 3 of diosgenin, mutagenesis of the amino acids predicted to interact with this position (Leu287, Arg367 and Tyr281) or derivatization (e.g., methylation) of the 3 hydroxyl group of diosgenin might be strategies worth pursuing in the future. Nevertheless, the information provided by the prediction of the underlying binding mode could already be used for further structural improvements in order to develop even more potent ROR inverse agonists on the basis of diosgenin. Of note, since the binding sites of RORγ and RORα are structurally highly conserved, it is plausible that diosgenin binds to both proteins with the same binding mode.

In addition to the aforementioned positive effects on Th17-mediated diseases, previous studies also found that diosgenin decreased plasma glucose levels in streptozotocin-induced diabetic rats [45]; reduced total cholesterol, triglyceride, and LDL levels in high-fat diet-fed Sprague–Dawley rats [46]; halted the progression of atherosclerosis by downregulation of pro-inflammatory mediators in Wistar rats [47]; and reduced the incidence of invasive and non-invasive colon tumors in azoxymethane-induced rats by up to 60% [48] (reviewed in [49,50]). These promising activities of diosgenin, however, are highly limited by the poor oral bioavailability of the compound, which precludes its potential therapeutic uses [51]. This issue could reportedly be overcome by employing various drug delivery strategies [52,53,54].

## 5. Conclusions

Taken together, in this study, we could demonstrate a direct inverse agonistic activity of diosgenin on the nuclear receptors RORα and RORγ for the first time. However, additional functional consequences of these activities on protein levels of distinct RORα/γ target genes, the experimental validation of the in silico predictions, as well as overcoming pharmacokinetic obstacles should be addressed in future studies.

**Figure 10:**
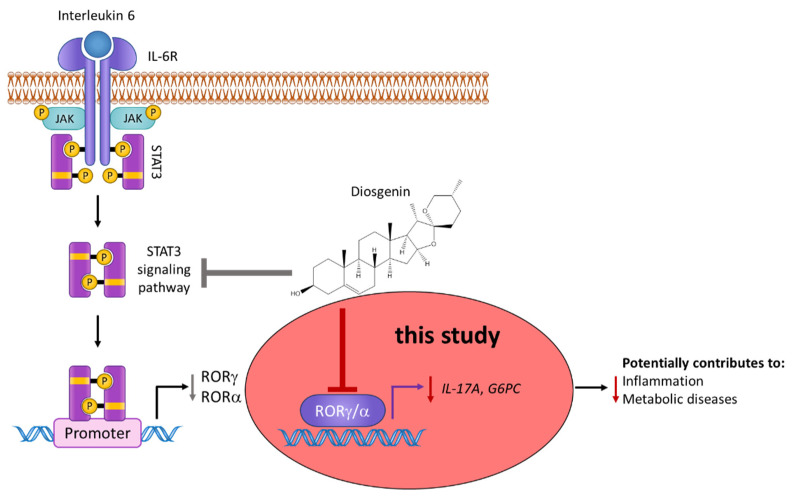
Overview on the updated pharmacological actions of diosgenin. Besides the already described effect of diosgenin on the expression and phosphorylation levels of STAT3 [42], this study now extends the activity profile of diosgenin to a direct and dual-specific modulation of the nuclear receptors RORα and RORγ (highlighted in red). The observed inverse agonistic activity on RORα/γ was further shown to significantly decrease the expression levels of the important ROR target genes *IL-17A* and *G6PC*, which are directly involved in the pathogenesis of Th17-driven inflammatory processes and metabolic processes and thereby potentially contribute to the beneficial effects of diosgenin observed in these conditions [55].

Collectively, our findings further extend the knowledge of the pharmacological profile of diosgenin and add to a more comprehensive picture of its beneficial effects in inflammatory and metabolic diseases reported elsewhere (reviewed in [55]) (Figure 10).

## Figures and Tables

**Table 1:** Primer used for RT-qPCR amplification.

Target Gene	Forward Primer	Reverse Primer
*IL-17A*	5′-ACCGATCCACCTCACCTTGG-3′	5′-AGTCCACGTTCCCATCAGCG-3′
*G6PC* [32]	5′-TCCATACTGGTGGGTTTTGG-3′	5′-GAGGAAAATGAGCAGCAAGG-3′
*GAPDH*	Hs_GAPDH_1_SG QuantiTect Primer Assay, GeneGlobe ID: QT00079247, Detected transcript: NM_001256799	
*beta actin*	Hs_ACTB_1_SG QuantiTect Primer Assay, GeneGlobe ID: QT00095431, Detected transcript: NM_001101	

## Data Availability

The data supporting the findings of this study are available from the corresponding author upon reasonable request.

## Supplementary Materials

The following supporting information can be downloaded at: https://www.mdpi.com/article/10.3390/biomedicines10092076/s1, Figure S1: Resazurin conversion assay of dioscin and protodioscin in HEK293 cells; Figure S2: Nuclear receptor-Gal4 luciferase assays of diosgenin in HEK293 cells; Figure S3: Resazurin conversion assay of diosgenin in Jurkat T and HepG2 cells; Table S1: Donated plasmids and providers; Table S2: Catalog numbers and providers of commercially obtained materials.

## Abbreviations

Ala (alanine); ANOVA (analysis of variance); Arg (arginine); ATCC (American Type Culture Collection); cDNA (complementary DNA); DMEM (Dulbecco’s modified Eagle medium); DNA (deoxyribonucleic acid); EDTA (ethylenediaminetetraacetic acid); eGFP (enhanced green fluorescent protein); EMEM (Eagle’s minimum essential medium); EtOH (ethanol); FBS (fetal bovine serum); FXR (farnesoid X receptor); G6PC (glucose-6-phosphatase); GAPDH (glyceraldehyde 3-phosphate dehydrogenase); HEK293 (human embryonic kidney 293); HepG2 (hepatoma G2); His (histidine); IC_50_ (half maximal inhibitory concentration); IL (interleukin); Ile (isoleucine); LBD (ligand-binding domain); Leu (leucine); Luc (luciferase); LXR (liver X receptor); Met (methionine); OPLS (optimized potentials for liquid simulations); PCR (polymerase chain reaction); Phe (phenylalanine); PPAR (peroxisome proliferator-activated receptor); RAR (retinoic acid receptor); RFU (relative fluorescence units); RLU (relative luminescence units); RMSD (root-mean-square deviation); RNA (ribonucleic acid); ROR (retinoic acid receptor-related orphan receptor); RORE (ROR response element); RPMI-1640 (Roswell Park Memorial Institute-1640 medium); RT-qPCR (reverse transcription quantitative PCR); RXR (retinoid X receptor); SD (standard deviation); STAT (signal transducer and activator of transcription); Th (T helper); tk (thymidine kinase); Tyr (tyrosine); Val (valine); λ_em_ (emission wavelength)

## References

[1] Solt L.A., Griffin P.R., Burris T.P. (2010). Ligand regulation of retinoic acid receptor-related orphan receptors: Implications for development of novel therapeutics. Curr. Opin. Lipidol..

[2] Stehlin C., Wurtz J.-M., Steinmetz A., Greiner E., Schüle R., Moras D., Renaud J.-P. (2001). X-ray structure of the orphan nuclear receptor RORβ ligand-binding domain in the active conformation. EMBO J..

[3] Ivanov I.I., McKenzie B.S., Zhou L., Tadokoro C.E., Lepelley A., Lafaille J.J., Cua D.J., Littman D.R. (2006). The orphan nuclear receptor RORgammat directs the differentiation program of proinflammatory IL-17+ T helper cells. Cell.

[4] Ivanov I.I., Zhou L., Littman D.R. (2007). Transcriptional regulation of Th17 cell differentiation. Semin. Immunol..

[5] Takeda Y., Kang H.S., Freudenberg J., DeGraff L.M., Jothi R., Jetten A.M. (2014). Retinoic acid-related orphan receptor gamma (RORgamma): A novel participant in the diurnal regulation of hepatic gluconeogenesis and insulin sensitivity. PLOS Genet..

[6] Guendisch U., Weiss J., Ecoeur F., Riker J.C., Kaupmann K., Kallen J., Hintermann S., Orain D., Dawson J., Billich A. (2017). Pharmacological inhibition of RORgammat suppresses the Th17 pathway and alleviates arthritis in vivo. PLoS ONE.

[7] Kumar N., Lyda B., Chang M.R., Lauer J.L., Solt L.A., Burris T.P., Kamenecka T.M., Griffin P.R. (2012). Identification of SR2211: A potent synthetic RORgamma-selective modulator. ACS Chem. Biol..

[8] Solt L.A., Kumar N., Nuhant P., Wang Y., Lauer J.L., Liu J., Istrate M.A., Kamenecka T.M., Roush W.R., Vidović D. (2011). Suppression of TH17 differentiation and autoimmunity by a synthetic ROR ligand. Nature.

[9] Jetten A.M., Kang H.S., Takeda Y. (2013). Retinoic acid-related orphan receptors alpha and gamma: Key regulators of lipid/glucose metabolism, inflammation, and insulin sensitivity. Front. Endocrinol..

[10] Yang X.O., Pappu B.P., Nurieva R., Akimzhanov A., Kang H.S., Chung Y., Ma L., Shah B., Panopoulos A.D., Schluns K.S. (2008). T helper 17 lineage differentiation is programmed by orphan nuclear receptors ROR alpha and ROR gamma. Immunity.

[11] Lau P., Fitzsimmons R.L., Raichur S., Wang S.C., Lechtken A., Muscat G.E. (2008). The orphan nuclear receptor, RORalpha, regulates gene expression that controls lipid metabolism: Staggerer (SG/SG) mice are resistant to diet-induced obesity. J. Biol. Chem..

[12] Kadiri S., Monnier C., Ganbold M., Ledent T., Capeau J., Antoine B. (2015). The nuclear retinoid-related orphan receptor-alpha regulates adipose tissue glyceroneogenesis in addition to hepatic gluconeogenesis. Am. J. Physiol. Endocrinol. Metab..

[13] Lau P., Fitzsimmons R.L., Pearen M.A., Watt M.J., Muscat G.E. (2011). Homozygous staggerer (sg/sg) mice display improved insulin sensitivity and enhanced glucose uptake in skeletal muscle. Diabetologia.

[14] Chopra A.R., Louet J.F., Saha P., An J., Demayo F., Xu J., York B., Karpen S., Finegold M., Moore D. (2008). Absence of the SRC-2 coactivator results in a glycogenopathy resembling Von Gierke’s disease. Science.

[15] Dong C. (2008). TH17 cells in development: An updated view of their molecular identity and genetic programming. Nat. Rev. Immunol..

[16] Huh J.R., Littman D.R. (2012). Small molecule inhibitors of RORgammat: Targeting Th17 cells and other applications. Eur. J. Immunol..

[17] Yang X., Downes M., Yu R.T., Bookout A.L., He W., Straume M., Mangelsdorf D.J., Evans R.M. (2006). Nuclear receptor expression links the circadian clock to metabolism. Cell.

[18] Kang H.S., Angers M., Beak J.Y., Wu X., Gimble J.M., Wada T., Xie W., Collins J.B., Grissom S.F., Jetten A.M. (2007). Gene expression profiling reveals a regulatory role for ROR alpha and ROR gamma in phase I and phase II metabolism. Physiol. Genom..

[19] Tai Lahans L. (2007). Integrating Conventional and Chinese Medicine in Cancer Care: A Clinical Guide.

[20] Yi T., Fan L.L., Chen H.L., Zhu G.Y., Suen H.M., Tang Y.N., Zhu L., Chu C., Zhao Z.Z., Chen H.B. (2014). Comparative analysis of diosgenin in Dioscorea species and related medicinal plants by UPLC-DAD-MS. BMC Biochem..

[21] Tsukamoto T., Ueno Y., Ohta Z. (1937). Diosgenin II. Glucoside of Dioscorea tokoro Makino. 3. Constitution of diosgenin. J. Pharm. Soc. Jpn..

[22] Marker R.E., Krueger J. (1940). Sterols. CXII. Sapogenins. XLI. The Preparation of Trillin and its Conversion to Progesterone. J. Am. Chem. Soc..

[23] Lemin A.J., Djerassi C. (1954). The Conversion of Diosgenin to Cortisone via 11-Ketosteroids of the 5β-Series. J. Am. Chem. Soc..

[24] Jesus M., Martins A.P., Gallardo E., Silvestre S. (2016). Diosgenin: Recent Highlights on Pharmacology and Analytical Methodology. J. Anal. Methods Chem..

[25] Kim J.K., Park S.U. (2018). An update on the biological and pharmacological activities of diosgenin. EXCLI J..

[26] Liu W., Zhu M., Yu Z., Yin D., Lu F., Pu Y., Zhao C., He C., Cao L. (2017). Therapeutic effects of diosgenin in experimental autoimmune encephalomyelitis. J. Neuroimmunol..

[27] Cao Y.J., Xu Y., Liu B., Zheng X., Wu J., Zhang Y., Li X.S., Qi Y., Sun Y.M., Wen W.B. (2019). Dioscin, a Steroidal Saponin Isolated from Dioscorea nipponica, Attenuates Collagen-Induced Arthritis by Inhibiting Th17 Cell Response. Am. J. Chin. Med..

[28] Xing E., Guo Y., Feng G., Song H., An G., Zhao X., Wang M. (2019). Effects of dioscin on T helper 17 and regulatory T-cell subsets in chicken collagen type II-induced arthritis mice. J. Chin. Med. Assoc..

[29] Song H., Gao Y., Wang Y., Guo Y., Xing E., Zhao X., Li W., Zhang J., Yu C. (2020). Effect of diosgenin on T-helper 17 cells in mice with collagen-induced arthritis. Pharmacogn. Mag..

[30] Wu S., Zhao M., Sun Y., Xie M., Le K., Xu M., Huang C. (2020). The potential of Diosgenin in treating psoriasis: Studies from HaCaT keratinocytes and imiquimod-induced murine model. Life Sci..

[31] Ladurner A., Schwarz P.F., Dirsch V.M. (2021). Natural products as modulators of retinoic acid receptor-related orphan receptors (RORs). Nat. Prod. Rep..

[32] Karas K., Salkowska A., Karwaciak I., Walczak-Drzewiecka A., Dastych J., Bachorz R.A., Ratajewski M. (2019). The Dichotomous Nature of AZ5104 (an EGFR Inhibitor) Towards RORgamma and RORgammaT. Int. J. Mol. Sci..

[33] Livak K.J., Schmittgen T.D. (2001). Analysis of relative gene expression data using real-time quantitative PCR and the 2(-Delta Delta C(T)) Method. Methods.

[34] Jones G., Willett P., Glen R.C., Leach A.R., Taylor R. (1997). Development and validation of a genetic algorithm for flexible docking. J. Mol. Biol..

[35] (2020). GOLD, *version v2020.2.0*.

[36] (2021). Maestro, *version 2021-3*.

[37] Roos K., Wu C., Damm W., Reboul M., Stevenson J.M., Lu C., Dahlgren M.K., Mondal S., Chen W., Wang L. (2019). OPLS3e: Extending Force Field Coverage for Drug-Like Small Molecules. J. Chem. Theory Comput..

[38] Kumar N., Solt L.A., Conkright J.J., Wang Y., Istrate M.A., Busby S.A., Garcia-Ordonez R.D., Burris T.P., Griffin P.R. (2010). The benzenesulfoamide T0901317 [N-(2,2,2-trifluoroethyl)-N-[4-[2,2,2-trifluoro-1-hydroxy-1-(trifluoromethyl)ethy l]phenyl]-benzenesulfonamide] is a novel retinoic acid receptor-related orphan receptor-alpha/gamma inverse agonist. Mol. Pharmacol..

[39] Hiebl V., Ladurner A., Latkolik S., Dirsch V.M. (2018). Natural products as modulators of the nuclear receptors and metabolic sensors LXR, FXR and RXR. Biotechnol. Adv..

[40] Xu T., Wang X., Zhong B., Nurieva R.I., Ding S., Dong C. (2011). Ursolic acid suppresses interleukin-17 (IL-17) production by selectively antagonizing the function of RORgamma t protein. J. Biol. Chem..

[41] Zhou Q., Qin S., Zhang J., Zhon L., Pen Z., Xing T. (2017). 1,25(OH)2D3 induces regulatory T cell differentiation by influencing the VDR/PLC-gamma1/TGF-beta1/pathway. Mol. Immunol..

[42] Li F., Fernandez P.P., Rajendran P., Hui K.M., Sethi G. (2010). Diosgenin, a steroidal saponin, inhibits STAT3 signaling pathway leading to suppression of proliferation and chemosensitization of human hepatocellular carcinoma cells. Cancer Lett..

[43] Wang R., Campbell S., Amir M., Mosure S.A., Bassette M.A., Eliason A., Sundrud M.S., Kamenecka T.M., Solt L.A. (2021). Genetic and pharmacological inhibition of the nuclear receptor RORalpha regulates TH17 driven inflammatory disorders. Nat. Commun..

[44] Manda V.K., Avula B., Ali Z., Wong Y.H., Smillie T.J., Khan I.A., Khan S.I. (2013). Characterization of in vitro ADME properties of diosgenin and dioscin from Dioscorea villosa. Planta Med..

[45] McAnuff M., Omoruyi F., Morrison E., Asemota H. (2005). Changes in some liver enzymes in streptozotocin-induced diabetic rats fed sapogenin extract from bitter yam (Dioscorea polygonoides) or commercial diosgenin. West. Indian Med. J..

[46] Li R., Liu Y., Shi J., Yu Y., Lu H., Yu L., Liu Y., Zhang F. (2019). Diosgenin regulates cholesterol metabolism in hypercholesterolemic rats by inhibiting NPC1L1 and enhancing ABCG5 and ABCG8. Biochim. Biophys. Acta Mol. Cell Biol. Lipids.

[47] Binesh A., Devaraj S.N., Halagowder D. (2018). Atherogenic diet induced lipid accumulation induced NFkappaB level in heart, liver and brain of Wistar rat and diosgenin as an anti-inflammatory agent. Life Sci..

[48] Malisetty V., Patlolla J., Raju J., Marcus L., Choi C., Rao C. (2005). Chemoprevention of colon cancer by diosgenin, a steroidal saponin constituent of fenugreek. Proc. Amer. Assoc. Cancer Res..

[49] Raju J., Rao C.V. (2012). Diosgenin, a steroid saponin constituent of yams and fenugreek: Emerging evidence for applications in medicine. Bioact. Compd. Phytomed..

[50] Wu F.C., Jiang J.G. (2019). Effects of diosgenin and its derivatives on atherosclerosis. Food Funct..

[51] Cayen M.N., Ferdinandi E.S., Greselin E., Dvornik D. (1979). Studies on the disposition of diosgenin in rats, dogs, monkeys and man. Atherosclerosis.

[52] Okawara M., Tokudome Y., Todo H., Sugibayashi K., Hashimoto F. (2014). Effect of beta-cyclodextrin derivatives on the diosgenin absorption in Caco-2 cell monolayer and rats. Biol. Pharm. Bull..

[53] Liu C.Z., Chang J.H., Zhang L., Xue H.F., Liu X.G., Liu P., Fu Q. (2017). Preparation and Evaluation of Diosgenin Nanocrystals to Improve Oral Bioavailability. AAPS PharmSciTech.

[54] Okawara M., Hashimoto F., Todo H., Sugibayashi K., Tokudome Y. (2014). Effect of liquid crystals with cyclodextrin on the bioavailability of a poorly water-soluble compound, diosgenin, after its oral administration to rats. Int. J. Pharm..

[55] Parama D., Boruah M., Yachna K., Rana V., Banik K., Harsha C., Thakur K.K., Dutta U., Arya A., Mao X. (2020). Diosgenin, a steroidal saponin, and its analogs: Effective therapies against different chronic diseases. Life Sci..

