# Supplementary material for: Identification of the Natural Steroid Sapogenin Diosgenin as a Direct Dual-Specific RORα/γ Inverse Agonist
Source: Biomedicines. 2022 Aug 25;10(9):2076. doi: 10.3390/biomedicines10092076 (PMC9495423; doi:10.3390/biomedicines10092076)
Supplement: Supplementary file 1 [file biomedicines-10-02076-s001.zip › biomedicines-1776875-supplementary.pdf]

Supplementary Materials

Identification of the natural steroid sapogenin diosgenin as a direct dual-specific RORα/γ inverse agonist

Patrik F. Schwarz, Alexander F. Perhal, Lucia N. Schöberl, Martin M. Kraus, Johannes Kirchmair, and Verena M. Dirsch

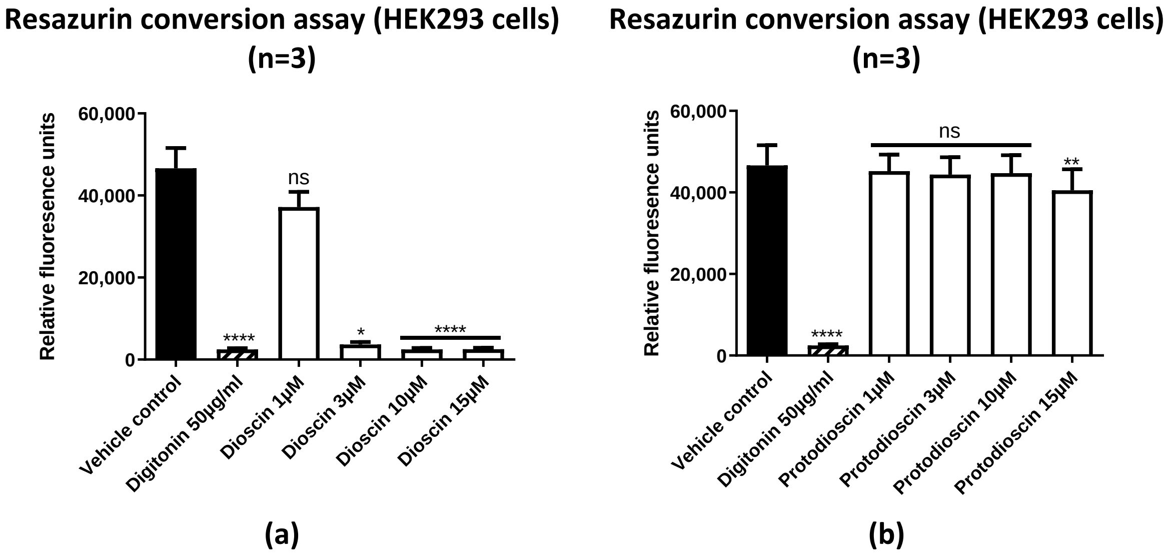

Figure S1. **Resazurin conversion assay of dioscin and protodioscin in HEK293 cells.** To exclude potential cytotoxic effects of dioscin and protodioscin in HEK293 cells, resazurin conversion assay were performed. Cells were treated with digitonin (50 µg/ml) as a positive control or dioscin/protodioscin at the indicated concentrations for 18 hours. After addition of resazurin (10 µg/ml) cells were incubated for another 5 hours before RFU values were measured at λ_{em} = 590 nm. Data are presented as means ± SD of three biological replicates (n=3) measured in technical quadruplicates. Kruskal-Wallis test followed by Dunn's post hoc test **(a)** or one-way ANOVA followed by Dunnett's post hoc test **(b)** were used for statistical analysis. ****p≤0.0001, **p≤0.01, *p≤0.05, ns p>0.05 as compared to vehicle control.

Nuclear receptor-Gal4 assays
(n=2-3)

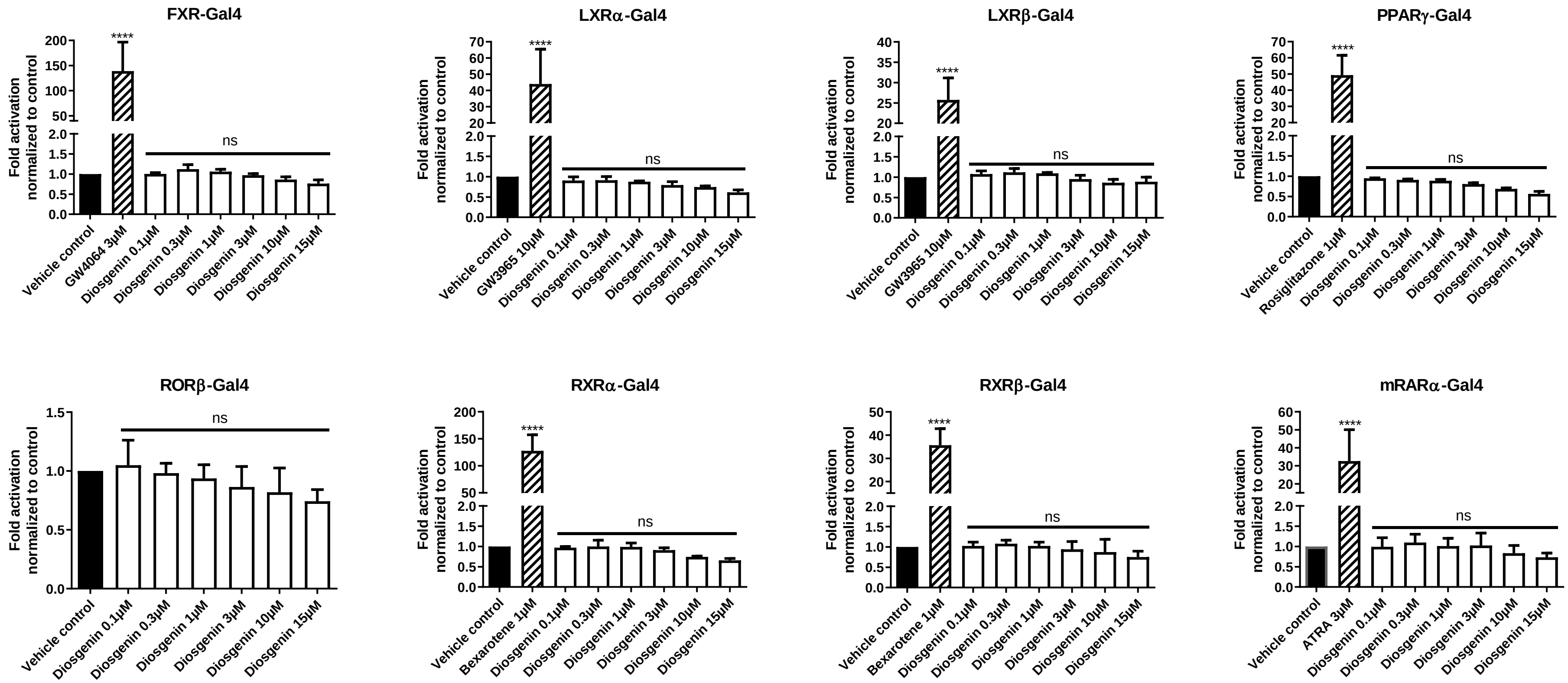

Figure S2. **Nuclear receptor-Gal4 luciferase assays of diosgenin in HEK293 cells.** Diosgenin was tested at different concentrations in cell-based nuclear receptor-Gal4 luciferase assays to determine its selectivity for RORγ and RORα. The luminescence signals derived from the luciferase reporter were normalized to eGFP fluorescence and expressed as fold activation normalized to the vehicle control (0.096% EtOH). Published agonists of the respective nuclear receptors were used as positive controls (except RORβ-Gal4, where a suitable positive control was not available). Bar charts represent transactivation activities expressed as mean ± SD of three biological replicates (n=3; except for GW3965 at LXRα-Gal4, n=2) measured in technical quadruplicates. One-way ANOVA followed by Dunnett's post hoc test were used for statistical analysis. **** p≤0.0001, ns p>0.05 as compared to vehicle control.

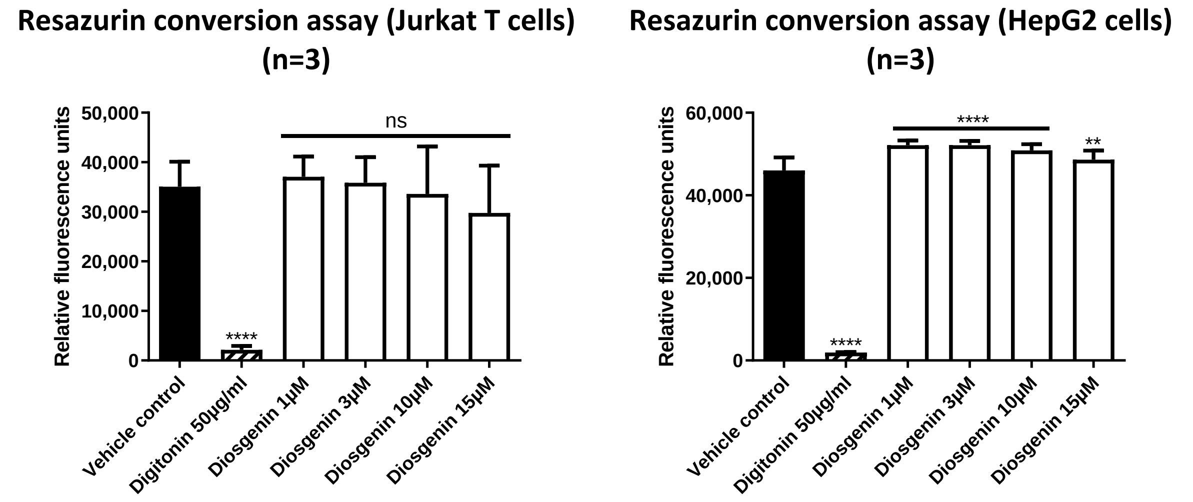

(a) **(b)**

Figure S3. **Resazurin conversion assay of diosgenin in Jurkat T and HepG2 cells.** To exclude potential cytotoxic effects of diosgenin in Jurkat T **(a)** and HepG2 cells **(b)**, a resazurin conversion assay was performed. Cells were treated with digitonin (50 µg/ml) as a positive control or diosgenin at the indicated concentrations for 18 hours. After addition of resazurin (10 µg/ml) cells were incubated for another 5 hours before RFU values were measured at λ_{em} = 590 nm. In HepG2 cells, diosgenin led to a slight but significant increase in fluorescence values. Data are presented as means ± SD of three biological replicates (n=3) measured in technical quadruplicates. one-way ANOVA followed by Dunnett's post hoc test was used for statistical analysis. ****$p \leq 0.0001$, **$p \leq 0.01$, ns $p > 0.05$ as compared to vehicle control.

Table S1. **Donated plasmids and providers.**

Plasmid	Provider
FXR-Gal4	Prof. Daniel Merk (Department of Pharmacy, Ludwig Maximilians University Munich, Munich, Bavaria, Germany)
LXRα/β-Gal4	Prof. Makoto Makishima (Nihon University School of Medicine, Tokyo, Kantō, Japan)
mRARα-Gal4, RXRβ-Gal4	Prof. Hinrich Gronemeyer (IGBMC: Institut de génétique, biologie moléculaire et cellulaire, Strasbourg, Grand Est, France)
PPARγ-Gal4, RXRα-Gal4, tk(MH1000)4xLuc	Prof. Ronald Evans (Salk Institute for Biological Studies, La Jolla, California, USA)
RORα-Gal4, RORγV1, RORE-Luc	Prof. Patrick Griffin (UF Scripps Biomedical Research, University of Florida, Jupiter, Flori-da, USA)
RORβ-Gal4	Prof. Laura A. Solt (UF Scripps Biomedical Research, University of Florida, Jupiter, Florida, USA)
RORγ-Gal4	Dr. Fabio R. Santori (Center for Molecular Medicine, University of Georgia, Athens, Georgia, USA)

Table S2. **Catalog numbers and providers of commercially obtained materials.**

Material	Catalog number	Provider
5X reporter lysis buffer	E3971	Promega
ATRA	R2625	Sigma-Aldrich
beta actin primer	249900	Qiagen
Bexarotene	SML0282	Sigma-Aldrich
Cell activation cocktail (without Brefeldin A)	423302	BioLegend
Digitonin	D141	Sigma-Aldrich
Dioscin	Cay11834	Biomol
Diosgenin	D1634	Sigma-Aldrich
DMEM	12-917F	Lonza
EDTA	8043.2	Carl Roth
EMEM	12-125F	Lonza
EtOH 96%	9065	Carl Roth
FBS	S1810 (batch number: S00CN)	biowest
G6PC primer	/	Microsynth
GAPDH primer	249900	Qiagen
GoTaq Green Master Mix	M712	Promega
GW3965	G6295	Sigma-Aldrich
GW4064	G5172	Sigma-Aldrich
HEK293 cells	CRL-1573	ATCC
HepG2 cells	HB-8065	ATCC
High-capacity cDNA Reverse Transcription Kit	4368814	Thermo Fisher Scientific
IL-17 primer	/	Microsynth
innuPREP RNA Mini Kit 2.0	845-KS-2040250	Analytik Jena
L-glutamine	BE17-605E	Lonza
Lipofectamine 3000	L3000001	Thermo Fisher Scientific
Lipofectamine LTX with PLUS Reagent	A12621	Thermo Fisher Scientific
pEGFP-N1	6085-1	Clontech
Penicillin-Streptomycin mixture	DE17-602E	Lonza
Protodioscin	Cay11887	Biomol
Resazurin sodium salt	199303	Sigma-Aldrich
Rosiglitazone	R2408	Sigma-Aldrich
RPMI-1640	12-167F	Lonza
SR2211	SML1170	Sigma-Aldrich
T0901317	T2320	Sigma-Aldrich
Trypsin	27250-018	Thermo Fisher Scientific
